# The Alzheimer's disease 5xFAD mouse model is best suited to investigate pretargeted imaging approaches beyond the blood-brain barrier

**DOI:** 10.3389/fnume.2022.1001722

**Published:** 2022-09-23

**Authors:** Sara Lopes van den Broek, Dag Sehlin, Jens V. Andersen, Blanca I. Aldana, Natalie Beschörner, Maiken Nedergaard, Gitte M. Knudsen, Stina Syvänen, Matthias M. Herth

**Affiliations:** ^1^ Department of Drug Design and Pharmacology, Faculty of Health and Medical Sciences, University of Copenhagen, Copenhagen, Denmark; ^2^ Rudbeck Laboratory, Department of Public Health and Caring Sciences, University of Uppsala, Uppsala, Sweden; ^3^ Center for Translational Neuromedicine, University of Copenhagen, Copenhagen, Denmark; ^4^ Neurobiology Research Unit and Center for Integrated Molecular Brain Imaging, Rigshospitalet Copenhagen University Hospital, Copenhagen, Denmark; ^5^ Department of Clinical Medicine, University of Copenhagen, Copenhagen, Denmark; ^6^ Department of Clinical Physiology, Nuclear Medicine / PET, Rigshospitalet Copenhagen University Hospital, Copenhagen, Denmark

**Keywords:** Alzheimer’s disease, amyloid beta, tetrazine ligation, pretargeted autoradiography, tetrazine, trans-cyclooctene, preclinical PET

## Abstract

Alzheimer's disease (AD) is the most common neurodegenerative disease, with an increasing prevalence. Currently, there is no ideal diagnostic molecular imaging agent for diagnosing AD. Antibodies (Abs) have been proposed to close this gap as they can bind selectively and with high affinity to amyloid β (Aβ)—one of the molecular hallmarks of AD. Abs can even be designed to selectively bind Aβ oligomers or isoforms, which are difficult to target with small imaging agents. Conventionally, Abs must be labeled with long-lived radionuclides which typically results in in high radiation burden to healthy tissue. Pretargeted imaging could solve this challenge as it allows for the use of short-lived radionuclides. To develop pretargeted imaging tools that can enter the brain, AD mouse models are useful as they allow testing of the imaging approach in a relevant animal model that could predict its clinical applicability. Several mouse models for AD have been developed with different characteristics. Commonly used models are: 5xFAD, APP/PS1 and tg-ArcSwe transgenic mice. In this study, we aimed to identify which of these models were best suited to investigate pretargeted imaging approaches beyond the blood brain barrier. We evaluated this by pretargeted autoradiography using the Aβ-targeting antibody 3D6 and an ^111^In-labeled Tz. Evaluation criteria were target-to-background ratios and accessibility. APP/PS1 mice showed Aβ accumulation in high and low binding brain regions and is as such less suitable for pretargeted purposes. 5xFAD and tg-ArcSwe mice showed similar uptake in high binding regions whereas low uptake in low binding regions and are better suited to evaluate pretargeted imaging approaches. 5xFAD mice are advantaged over tg-ArcSwe mice as pathology can be traced early (6 months compared to 18 months of age) and as 5xFAD mice are commercially available.

## Introduction

**Figure 1:**
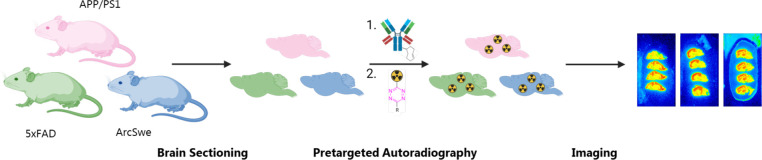
Schematic overview of the study outline. Brains from APP/PS1, 5xFAD and tg-ArcSwe mice are sectioned and tested by pretargeted autoradiography to compare the imaging contrast between these mouse models.

Alzheimer's disease (AD) is the most common neurodegenerative disease, affecting nearly 50 million people worldwide with expenses in the order of more than 1% of the global economy (1, 2). Higher prevalence is expected in the coming decades due to ageing populations worldwide (3, 4). AD is characterized by a spectrum of clinical symptoms. The most prominent and significant symptoms are progressive memory loss and decline in cognitive function (5). Up until today, the exact biological mechanisms underlying AD are unclear. However, cellular hallmarks causing AD pathology have been defined. Extracellular accumulation of amyloid beta (Aβ) plaques plays a crucial role in disease onset and progress (5, 6). More specifically, the 38- to 42-aminoacid Aβ peptides are the drivers of senile plaques formation (7). The amyloid hypothesis, stating that cerebral Aβ accumulation is the main driver of AD pathology, led to the development of several anti-Aβ therapies aiming to image, prevent and treat AD. Therapeutic antibodies (Abs) targeting different Aβ peptides aim at reducing the overall Aβ aggregation rate and amount of Aβ aggregation in the brain. However, increasing evidence shows that different Aβ aggregates cause diverse pathologies, and therefore high specificity towards these specific Aβ forms is a necessity for adequate and accurate drug treatment and imaging of AD at an early stage (1). In general, drug development for AD has been stagnant over the past decades, which may be related to a lack of biomarker tools. For many therapeutic targets, it remains impossible to measure target engagement or impact on disease progression—primarily due to the lack of suitable Positron emission tomography (PET) ligands. PET is a non-invasive nuclear molecular imaging method that allows visualization and quantification of biochemical processes *in vivo*. This technique relies on target-specific radioactive molecules—radioligands—that are administered to the patient prior to scanning (8–10). Abs have excellent target specificity which, combined with improved brain penetration, e.g., by conjugating a transferrin receptor (TfR) targeting moiety, may serve as an alternative approach to address these challenges. Preclinical data suggest that Ab-based PET imaging of Aβ plaques is feasible (11). However, their slow pharmacokinetics pose a major challenge, with respect to signal-to-noise ratios, as well as patient radiation burden to healthy tissue. This may be overcome by pretargeted imaging based on *in vivo* labeling of a target-bound large molecule with a small molecule tracer (12). Pretargeted imaging is a two-step approach where a tagged Ab is administered first, allowing accumulation at its target and clearance from blood circulation, before a second small radioactive molecule is administered that reacts bioorthogonally with the tagged Ab. Consequently, the pretargeted Ab can be radiolabeled *in vivo* and, the target of the Ab imaged with short-lived radionuclides such as fluorine-18, the most clinically relevant radionuclide. Currently, the most attractive reaction for pretargeted brain imaging is the tetrazine ligation. This is due to the high selectivity and fast reaction kinetics of this ligation (>50,000 M^−1^s^−1^), that is typically based on the bioorthogonal reaction between tetrazines (Tzs) and a *trans*-cyclooctene (TCOs) (13–16). However, pretargeted imaging beyond the blood-brain barrier (BBB) has not been achieved thus far. As pretargeting beyond the BBB is dependent at least seven factors [(1) possibility of the mAb to cross the BBB, (2) *in vivo* stability of the TCO in the brain, (3) BBB permeability and reactivity of the Tzs, (4) concentration of the target (the animal model), (5) metabolism of the Tzs, (6) concentration of the pretargeting vector and the Tz as well as (7) the clearance of the mAb from the blood pool before the tetrazine is injected], it makes sense to reduce the challenge first to single parameter. In this manuscript, we study which animal model is most suited (Figure 1).

In this study, three different AD mouse models—5xFAD, APP/PS1 and tg-ArcSwe—were evaluated with respect to feasibility as models for pretargeted approaches. Evaluation criteria were target-to-background ratios and accessibility of the model. We investigated these criteria using *in vitro* pretargeted autoradiography using the Aβ-targeting antibody 3D6. Using this methodology, the two-step pretargeting approach could be investigated in the three animal models to give insights in the preferred animal model of choice for *in vivo* pretargeting. For these studies, we used the the highly polar 1,4,7,10-tetraazacyclododecane-1,4,7,10-tetraacetic acid (DOTA)-PEG_11_-tetrazine radiolabeled with indium-111 due to its low non-displaceable binding and fast kinetics towards the TCOs (16).

### Mouse models of AD

Despite substantial efforts into developing appropriate mouse models of late onset AD (LOAD), there is currently no single model that covers all the features of human AD (17). After the identification of the APP gene mutation as a major contributor to AD onset, initial transgenic (Tg) mouse models express mainly human APP (hAPP) (3, 5). The most commonly used mutation is the Swedish APP mutation (K670N/M671l) which causes overproduction of total Aβ from APP. Tg-APP mice represent only a part of AD pathology and therefore combinations of other mutant mice have been developed. Nowadays, there are also mouse models mimicking other human genomic alterations, including PS1 mutations that have been combined with APP to create the APP/PS1 double transgenic model (18). Aβ pathology in sporadic and familial cases are largely morphologically similar, and therefore mouse models with genetically engineered familial AD (FAD) mutations can be rationalized for understanding sporadic LOAD (3). It is critical to consider the potential mechanism of Aβ accumulation when developing and using a mouse APP model. For example, in FAD, Aβ deposition is either caused by increased production of Aβ; by intra-Aβ sequence mutations that alter its structural properties; or by increased Aβ production due to increased enzymatic cleavage of APP at the β-site (19). In the following, we describe briefly the three different mouse models used in this study.

#### 5xFAD

The 5xFAD mouse model was generated in the Vassar laboratory and is one of the most used AD models (5, 20). This model was developed to accelerate amyloid deposition *in vivo* and contains five combinatorial mutations (Swedish K670N/M671l, London V717I and Florida I716V in hAPP, M146l and L286V in PS1). These synergistic mutations lead to fast disease progression. 5xFAD mice show high expression of cerebral Aβ42 after 1.5 months of age, and amyloid deposition and gliosis becomes evident already after 2 months. Amyloid deposition at 9 months of age results in neurodegeneration and neuronal loss (20). Next to this, the neuroimaging characterization of this model is still incomplete due to a scarcity of publications that characterize and phenotype this mouse model (5).

#### APP/PS1

The APP/PS1 line are double transgenic mice expressing a chimeric mouse/human amyloid precursor protein (Mo/HuAPP695swe) and a mutant human presenilin 1 (PS1-dE9). Both mutations are associated with early-onset Alzheimer's disease (21, 22). The Mo/HuAPP695swe transgene allows the mice to secrete a human Aβ peptide. The included Swedish mutations (K595N/M596l) elevate the amount of Aβ produced from the transgene by favoring processing through the beta-secretase pathway (23). APP/PS1 mice reproduce some of the neuropathological and cognitive deficits that are observed in human AD. Reports detail that transgenic mice develop cerebral amyloid deposits by 6 months of age, with abundant plaques in the hippocampus and cortex by 9 months. Between 6 and 15 months of age, mice exhibit a gender-based disparity in Aβ burden. Females develop a 5-fold (Aβ42) and 10-fold (Aβ40) increase in Aβ deposits in the cerebellum by 15 months as compared to males. Accumulation of plaques is more abundant in the molecular layer than in the granular layer. In cortex, the amyloid burden is increased in both sexes in parallel. Astrocytosis develops in parallel with plaque deposition, with severe gliosis starting around 6 months, especially in the vicinity of plaques. The number of glial fibrillary acidic protein (GFAP)-positive cells progressively increases with age, with extensive staining throughout the cortex by 15 months. Between 8 and 10 months, modest neuronal loss has been observed adjacent to plaques relative to more distal areas (24).

#### Tg-ArcSwe

Tg-ArcSwe mice carry both the Arctic mutation, which is located within the Aβ domain of the APP gene and facilitates Aβ protofibril formation, and the previously mentioned Swedish mutation, which increases generation of Aβ peptides. The Swedish mutation accelerates the age onset in the mice compared to animals with only the Arctic mutation. The tg-ArcSwe model has a marked phenotype with age-dependent intraneuronal Aβ accumulation occurring several months before the onset of extracellular plaque formation. This has also been observed in post-mortem human AD brains. Morphological studies have shown progressive structural changes of plaque formation in combination with dynamic response in surrounding tissue, e.g., swollen and distorted dendrites and inflammatory reactions, starting when the mice are 6 months of age (25–27). After 12 months of age, Aβ40 and Aβ42 plaque deposits are consistently present in the cerebral cortex, hippocampus and thalamus. Gender comparison studies have revealed that there is a lower plaque load in the hippocampus of female mice, but no differences were observed in other regions (28).

## Results and discussion

### Pretargeted imaging using the tetrazine ligation

**Figure 2:**
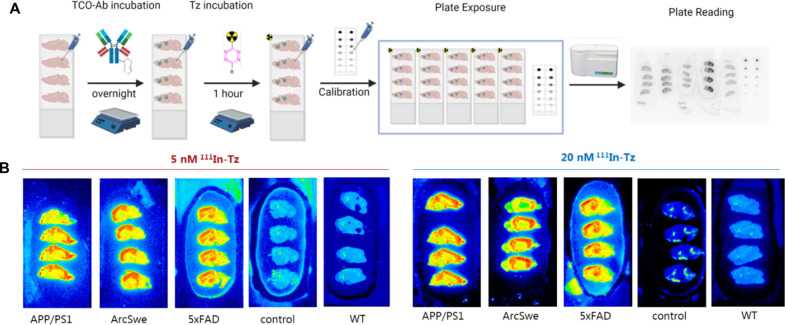
(**A**) Schematic overview of pretargeted autoradiography. Control = Tg mice brains that received only ^111^In-Tz (no TCO-mAb). WT animals received TCO-3D6 and ^111^In-Tz. (**B**) Representative pretargeted autoradiography images at 0.06 µg/ml TCO-3D6.

Pretargeting is based on a two-step approach. Success is determined by the properties of each agent used in both steps. We have recently developed a ^18^F-labeling strategy (29) that allows to label tetrazine based imaging agents capable of entering the brain and react with targets beyond the BBB (16). We also estimated the possibility to use existing Ab trafficking approaches to reach brain TCO concentrations suitable for pretargeted imaging beyond the BBB. Required concentration are estimated to be reachable. In this study, our aim was to evaluate which AD mouse model is best suited to test the feasibility of pretargeted brain imaging. For this purpose, we determined target-to-background ratios of high-to-low binding regions in three AD models. Pretargeted imaging was used to determine these ratios (Figure 2A) (30, 31). We evaluated the imaging contrast using a TCO modified monospecific mAb 3D6 (32) that targets Aβ and an ^111^In-labeled tetrazine that has already been successfully used for pretargeted imaging (16). 3D6 was non-site-specifically modified with TCO-PEG_4_-NHS to give an average of approximately 11 TCOs/Ab (Supplementary Material).

Sagittal sections of AD or corresponding WT mouse brains (APP/PS1, tg-ArcSwe, 5xFAD and WT) were prepared. In accordance to the general pretargeted autoradiography workflow (16), TCO-3D6 was applied first. Two different TCO-Ab concentrations were investigated: 0.006 and 0.06 μg/ml. After 24 h incubation, an ^111^In-labeled Tz [1,4,7,10-tetraazacyclododecane-1,4,7,10-tetraacetic acid (DOTA)-PEG_11_-tetrazine] was used for imaging purposes. Two different ^111^In-Tz concentrations—5 and 20 nM—were used to evaluate the binding. Tg mice received only ^111^In-Tz and WT mice were used as controls. Tetrazine binding was determined in the cerebral cortex (Aβ high binding region) and in the cerebellum (Aβ low binding region) (5, 28, 33). Representative examples of obtained pretargeted autoradiography results are visualized in Figure 2.

### Comparison APP/PS1, tg-ArcSwe and 5xFAD

**Figure 3:**
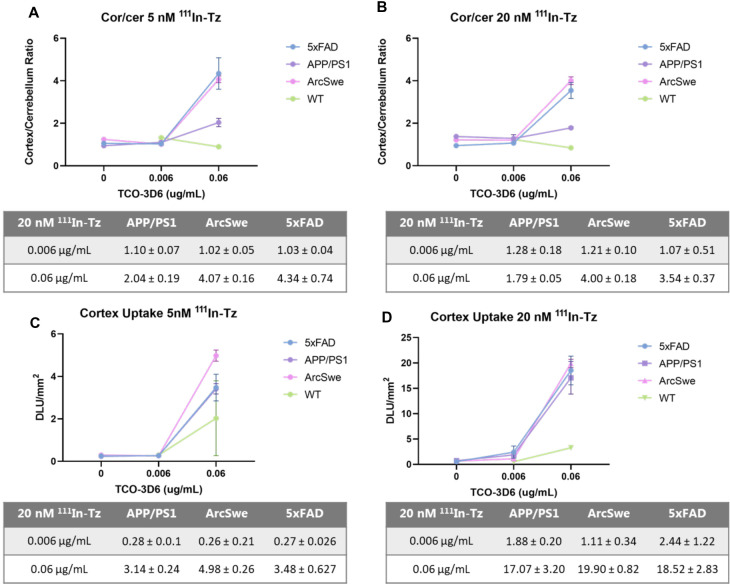
Cortex/cerebellum ratio and cortex uptake in 5xFAD, APP/PS1, tg-ArcSwe and WT mice determined by pretargeted autoradiography. (**A**) Cor/cer ratio using 5 nM ^111^In-Tz (*n* = 8 per mouse model). (**B**) Cor/cer ratio using 20 nM ^111^In-Tz (*n* = 8 per mouse model). (**C**) Cortex uptake using 5 nM ^111^In-Tz (*n* = 8 per mouse model). (**D**) Cortex uptake using 20 nM ^111^In-Tz (*n* = 8 per mouse model).

Region-of-interest (ROIs) were manually mapped around the cerebral cortex (cor) and the cerebellum (cer) and were used to calculate the cor/cer ratio (Figures 3A,B). Similar accumulations and ratios were observed when applying 5 or 20 nM ^111^In-Tz (Figure 1). Pretargeted autoradiography in 5xFAD and tg-ArcSwe mice resulted in similar cor/cer ratios of approx. 4 using 0.06 µg/ml TCO-3D6 (Figure 3). A clear imaging contrast was visual using 0.06 μg/ml of TCO-3D6. APP/PS1 mice, on the other hand, showed a much lower cor/cer ratio of 2. As previously mentioned, APP/PS1 mice have pathology in different brain tissues, including the cerebellum. It has also been shown previously that models with extensive cerebellum pathology, including models that harbor PS1 mutations, show *in vivo* amyloid-PET cor/cer ratios close to unity and that the animal model is crucial when ratios are used as the main read-out. However, the absolute uptake in the cortex was similar in all animal models (Figures 3C,D). As expected, a higher overall uptake was observed using 20 nM ^111^In-Tz.

## Conclusion

This study describes the comparison and evaluation of three different AD mouse models—5xFAD, APP/PS1 and tg-ArcSwe—by pretargeted autoradiography using mAb TCO-3D6 and ^111^In-Tz. All mouse models showed comparable uptake in the cerebral cortex. APP/PS1 mice also showed cerebellar uptake, resulting in a lower cortex/cerebellum ratio compared to the other two models. Both absolute uptake and cor/cer ratio of 5xFAD and ArcSwe were similar and both models are therefore suitable for pretargeted PET studies. 5xFAD mice are commercially available and develop pathology at an early age—we used animals of 6 months of age compared to 18 months of age for the tg-ArcSwe mice—which might be beneficial for practical reasons.

## Data Availability

The original contributions presented in the study are included in the article/**Supplementary Material**, further inquiries can be directed to the corresponding author/s.

## References

[1] Linse S Scheidt T Bernfur K Vendruscolo M Dobson CM Cohen SIA Kinetic fingerprints differentiate the mechanisms of action of anti-Aβ antibodies. Nat Struct Mol Biol. (2020) 27:1125–33. 10.1038/s41594-020-0505-6 32989305

[2] Ronaldson PT Davis TP. Regulation of blood–brain barrier integrity by microglia in health and disease: a therapeutic opportunity. J Cereb Blood Flow Metab. (2020) 40:S6–S24. 10.1177/0271678X20951995 32928017 PMC7687032

[3] Sasaguri H Nilsson P Hashimoto S Nagata K Saito T De Strooper B APP mouse models for Alzheimer’s disease preclinical studies. EMBO J. (2017) 36:2473–87. 10.15252/embj.201797397 28768718 PMC5579350

[4] Winblad B Amouyel P Andrieu S Ballard C Brayne C Brodaty H Defeating Alzheimer’s disease and other dementias: a priority for European science and society. Lancet Neurol. (2016) 15:455–532. 10.1016/S1474-4422(16)00062-4 26987701

[5] Jullienne A Trinh MV Obenaus A. Neuroimaging of mouse models of Alzheimer’s disease. Biomedicines. (2022) 10:1–35. 10.3390/biomedicines10020305 PMC8869427 35203515

[6] Serrano-Pozo A Frosch MP Masliah E Hyman BT. Neuropathological alterations in Alzheimer disease. Cold Spring Harb Perspect Med. (2011) 1:1–24. 10.1101/cshperspect.a006189 PMC3234452 22229116

[7] Elder GA Gama Sosa MA De Gasperi R. Transgenic mouse models of Alzheimer’s disease. Mt Sinai J Med. (2010) 77:69–81. 10.1002/msj.20159 20101721 PMC2925685

[8] Zanzonico P. Positron emission tomography: a review of basic principles, scanner design and performance, and current systems. Semin Nucl Med. (2004) 34:87–111. 10.1053/j.semnuclmed.2003.12.002 15031810

[9] Ametamey SM Honer M Schubiger PA. Molecular imaging with PET. Chem Rev. (2008) 108:1501–16. 10.1021/cr0782426 18426240

[10] Basu S Kwee TC Surti S Akin EA Yoo D Alavi A. Fundamentals of PET and PET/CT imaging. Ann N Y Acad Sci. (2011) 1228:1–18. 10.1111/j.1749-6632.2011.06077.x 21718318

[11] Sehlin D Fang XT Cato L Antoni G Lannfelt L Syvänen S. Antibody-based PET imaging of amyloid beta in mouse models of Alzheimer’s disease. Nat Commun. (2016) 7:1–11. 10.1038/ncomms10759 PMC4762893 26892305

[12] Rossin R Robillard MS. Pretargeted imaging using bioorthogonal chemistry in mice. Curr Opin Chem Biol. (2014) 21:161–9. 10.1016/j.cbpa.2014.07.023 25159021

[13] Stéen EJL Edem PE Nørregaard K Jørgensen JT Shalgunov V Kjaer A Pretargeting in nuclear imaging and radionuclide therapy: improving efficacy of theranostics and nanomedicines. Biomaterials. (2018) 179:209–245. 10.1016/j.biomaterials.2018.06.021 30007471

[14] Rossin R Van Duijnhoven SMJ Läppchen T Van Den Bosch SM Robillard MS. Trans-cyclooctene tag with improved properties for tumor pretargeting with the Diels-Alder reaction. Mol Pharm. (2014) 11:3090–6. 10.1021/mp500275a 25077373

[15] Wang M Vannam R Lambert WD Xie Y Wang H Giglio B Hydrophilic 18 F-labeled trans-5-oxocene (oxoTCO) for efficient construction of PET agents with improved tumor-to-background ratios in neurotensin receptor (NTR) imaging. Chem Commun. (2019) 55:2485–8. 10.1039/C8CC09747J PMC7347157 30735213

[16] Shalgunov V Lopes van den Broek S Anderson IV Vázquez RG Raval N Palner M Pretargeted imaging beyond the blood-brain barrier. ChemRxiv. (2022) 1–12. [preprint]. 10.26434/chemrxiv-2022-gj597-v2

[17] Vitek MP Araujo JA Fossel M Greenberg BD Howell GR Sukoff Rizzo SJ Translational animal models for Alzheimer’s disease: an Alzheimer’s association business consortium think tank. Alzheimer’s Dement Transl Res Clin Interv. (2020) 6:1–12. 10.1002/trc2.12114 PMC7798310 33457489

[18] Jankowsky JL Fadale DJ Anderson J Xu GM Gonzales V Jenkins NA Mutant presenilins specifically elevate the levels of the 42 residue β-amyloid peptide in vivo: evidence for augmentation of a 42-specific *γ* secretase. Hum Mol Genet. (2004) 13:159–70. 10.1093/hmg/ddh019 14645205

[19] Selkoe DJ Hardy J. The amyloid hypothesis of Alzheimer’s disease at 25 years. EMBO Mol Med. (2016) 8:595–608. 10.15252/emmm.201606210 27025652 PMC4888851

[20] Oakley H Cole SL Logan S Maus E Shao P Craft J Intraneuronal beta-amyloid aggregates, neurodegeneration, and neuron loss in transgenic mice with five familial Alzheimer’s disease mutations: potential factors in amyloid plaque formation. J Neurosci. (2006) 26:10129–40. 10.1523/JNEUROSCI.1202-06.2006 17021169 PMC6674618

[21] Blanchard V Moussaoui S Czech C Touchet N Bonici B Planche M Time sequence of maturation of dystrophic neurites associated with Aβ deposits in APP/PS1 transgenic mice. Exp Neurol. (2003) 184:247–63. 10.1016/S0014-4886(03)00252-8 14637096

[22] Delatour B Guégan M Volk A Dhenain M. In vivo MRI and histological evaluation of brain atrophy in APP/PS1 transgenic mice. Neurobiol Aging. (2006) 27:835–47. 10.1016/j.neurobiolaging.2005.04.011 16023262

[23] Trinchese F Liu S Battaglia F Walter S Mathews PM Arancio O. Progressive age-related development of Alzheimer-like pathology in APP/PS1 mice. Ann Neurol. (2004) 55:801–14. 10.1002/ana.20101 15174014

[24] González-Domínguez R García-Barrera T Vitorica J Gómez-Ariza JL. Region-specific metabolic alterations in the brain of the APP/PS1 transgenic mice of Alzheimer’s disease. Biochim Biophys Acta Mol Basis Dis. (2014) 1842:2395–402. 10.1016/j.bbadis.2014.09.014 25281826

[25] Lord A Kalimo H Eckman C Zhang X-Q Lannfelt L Nilsson LNG. The arctic Alzheimer mutation facilitates early intraneuronal Aβ aggregation and senile plaque formation in transgenic mice. Neurobiol Aging. (2006) 27:67–77. 10.1016/j.neurobiolaging.2004.12.007 16298242

[26] Lord A Englund H Söderberg L Tucker S Clausen F Hillered L Amyloid-β protofibril levels correlate with spatial learning in arctic Alzheimer’s disease transgenic mice. FEBS J. (2009) 276:995–1006. 10.1111/j.1742-4658.2008.06836.x 19215301 PMC2752010

[27] Philipson O Hammarström P Nilsson KPR Protelius E Olofsson T Ingelsson M A highly insoluble state of Aβ similar to that of Alzheimer’s disease brain is found in arctic APP transgenic mice. Neurobiol Aging. (2009) 30:1393–405. 10.1016/j.neurobiolaging.2007.11.022 18192084

[28] Lillehaug S Syverstad GH Nilsson LNG Bjaalie JG Leergaard TB Torp R. Brainwide distribution and variance of amyloid-beta deposits in tg-ArcSwe mice. Neurobiol Aging. (2014) 35:556–64. 10.1016/j.neurobiolaging.2013.09.013 24126157

[29] García-Vázquez R Battisti UM Jørgensen JT Shalgunov V Hvass L Stares DL Direct Cu-mediated aromatic18F-labeling of highly reactive tetrazines for pretargeted bioorthogonal PET imaging. Chem Sci. (2021) 12:11668–75. 10.1039/D1SC02789A 34659701 PMC8442695

[30] Griem-Krey N Klein AB Herth M Wellendorph P. Autoradiography as a simple and powerful method for visualization and characterization of pharmacological targets. J Vis Exp. (2019) 12(145):1–11. 10.3791/58879 30933077

[31] Patel S Hamill T Hostetler E Burns HD Gibson RE. An in vitro assay for predicting successful imaging radiotracers. Mol Imaging Biol. (2003) 5:65–71. 10.1016/S1536-1632(03)00041-6 14499146

[32] Fang XT Hultqvist G Meier SR Antoni G Sehlin D Syvänen S. High detection sensitivity with antibody-based PET radioligand for amyloid beta in brain. Neuroimage. (2019) 184:881–8. 10.1016/j.neuroimage.2018.10.011 30300753

[33] López-González I Schlüter A Aso E Garcia-Esparcia P Ansoleaga B LLorens F Neuroinflammatory signals in Alzheimer disease and APP/PS1 transgenic mice: correlations with plaques, tangles, and oligomeric species. J Neuropathol Exp Neurol. (2015) 74:319–44. 10.1097/NEN.0000000000000176 25756590

