# Supplementary material for: The Alzheimer's disease 5xFAD mouse model is best suited to investigate pretargeted imaging approaches beyond the blood-brain barrier
Source: Front Nucl Med. 2022 Sep 23;2:1001722. doi: 10.3389/fnume.2022.1001722 (PMC11466232; doi:10.3389/fnume.2022.1001722)
Supplement: Supplementary file 1 [file Datasheet1.docx]

Supplementary Information

Table of Contents

[Section S1: Antibody modification with TCO-PEG_4_-NHS ester 2](#_Toc102322423)

[Section S2: Quantification TCO-loading Antibody 2](#_Toc102322424)

[Section S3: Radiochemistry 2](#_Toc102322425)

[General information 2](#_Toc102322426)

[^111^In-labeling Tz 2](#_Toc102322427)

[Section S4: Transgenic mice 3](#_Toc102322428)

[APP/PS1 3](#_Toc102322429)

[5xFAD mice 3](#_Toc102322430)

[ArcSwe mice 4](#_Toc102322431)

[Pretargeted Autoradiography 4](#_Toc102322432)

[References 4](#_Toc102322433)

# Section S1: Antibody modification with TCO-PEG_4_-NHS ester

Lysine residues in mAb 3D6 and mA3D6scFv8D3 were functionalized with TCO-PEG_4_-NHS ester.^1^ Sodium carbonate buffer (3.1 μL, 1 M, pH 8.0) was added to PBS containing mAb 3D6 (100 μL. 1.0 mg/mL), followed by addition of equatorial TCO-PEG_4_-NHS (Broadpharm, BP-22418) in acetonitrile using 100 eq. TCO−antibody ligand molar ratio. The mixture incubated at 600 rpm for 2 hours at room temperature in the dark. The solution was purified to remove the unreacted TCO-PEG_4_-NHS ester with Zeba spin desalting columns (7K MWCO, 0.5 mL, 89882, Thermofisher) and eluted in PBS (pH 7.4). Final protein concentration was measured with Nanodrop (NanoDrop 2000, ThermoScientific).

# Section S2: Quantification TCO-loading Antibody

Titration experiments were conducted to quantify the amount of reactive TCOs per protein-conjugate according to previously reported procedure.^2^ Aliquots of TCO-mAb in PBS (5 µL of 0.1 mg/mL solutions) were mixed with aliquots of ^111^In-labeled Tz ([^111^In]**1)** stock (5 µL, SI, Section S4) containing 2-3 eq. of Tz per expected amount of TCOs on the TCO-mAbs, and the mixed samples were incubated at 600 rpm for 1 hour at 37 °C. 3 μL of NuPAGE™ LDS Sample Buffer (NP0007, Invitrogen) was added and the mixture was shaken for 10 minutes at 70 °C. Samples were applied to NuPAGE™ 4 to 12%, Bis-Tris, 1.0 mm, Mini Protein Gel, 12-well (NP0322BOX , Invitrogen) SDS-PAGE gels. SDS-PAGE gels were exposed to phosphor storage screens and read by a Cyclone Storage Phosphor System (PerkinElmer Inc.). Quantification of plate readings was done with Optiquant software (version 5.00, PerkinElmer Inc.), showing an average of 11 TCOs/Ab.

# Section S3: Radiochemistry

## General information

Radiochemistry was performed at the Department of Clinical Physiology, Nuclear Medicine and PET, Rigshospitalet, Denmark. Unless otherwise stated, all reagents and solvents were purchased from commercial suppliers and used without further purification. [^111^In]InCl_3_ (non-carrier added) was purchased from Curium. 1,4,7,10-tetraazacyclododecane-1,4,7,10-tetraacetic acid (DOTA) -PEG_11_-tetrazine was received from Tagworks Pharmaceuticals. All the water used was ultrapure (> 18.2 MΩ cm^-1^) and metalfree. Other solvents were analytical or HPLC grade and used as received. The analytical-HPLC system consists of a 170U UVD detector, a Scansys radiodetector and a Dionex system connected to a P680A pump. The system was run by Chromeleon software.

## ^111^In-labeling Tz

The ^111^In-labeling was performed as previously described (Scheme S1).^2^ In general, **1** was dissolved (2 mg/mL) in metal-free water and stored at -80 °C before use. An aliquot of 50-100 μL (10-30 MBq) of [111In]indium chloride in 0.05 M HCl was combined with 2 μL **1** and 1 M NH_4_OAc buffer (pH 5.5) at a volume ratio of 1:10 was added. The mixture was shaken at 600 rpm for 5 min at 60 °C in an Eppendorf ThermoMixer C. Then, 10 mM diethylenetriamine-pentaacetic acid DTPA (volume ratio 1:10) and 2 μL 10 mg/mL gentisic acid in saline was added and the solution was shaken for an additional 5 min at 60 °C in an Eppendorf ThermoMixer C. Typically, a quantitative labeling yield and a radiochemical purity (RCP) > 95% were obtained with this method, as confirmed by radio-HPLC (Aeris Peptide C18-XB 3.6 µm 150x4.6 mm column. Solvent A = 0.1% TFA in water, solvent B = 0.1% TFA in acetonitrile. HPLC elution method: 0-1 min – 5% B, 1-8 min - gradient from 5% B to 75% B, 8-9 min – 75% B, 9-9.5 min - back to 5% B, 9.5-10 min – 5% B; flow rate 1.5 mL/min).

*Scheme S1. Preparation of [^111^In]3. i) ^111^InCl, 1M NH4OAc, 60 ºC, 5 min ii) 10 mM DTPA, gentisic acid, 60 ºC, 5 min*

# Section S4: Transgenic mice

## APP/PS1

Transgenic heterozygote male APP/PS1 (B6C3-Tg(APPswe,PSEN1dE9)85Dbo/Mmjax) have been purchased from Jackson Laboratories (Bar Harbor, ME, USA), strain: #034832-JAX. Breeding colonies were maintained at Panum, AEM Unit 16.2, University of Copenhagen. Animals were group housed in ventilated cages and observed every second day, as minimum, for any sign of distress/discomfort, weight loss, or infections. Mice were housed together in individually ventilated cages in a specific pathogen-free, humidity and temperature-controlled facility (12h light/dark cycle) with free access to water and chow. Mice were deeply anesthesized with Ketamin (80−140 mg/kg)/Xylazine (5−15 mg/kg) anesthesia and transcardially perfused with ice cold saline solution at a rate of 5ml/min. Afterwards, the brains were extracted and immediately frozen & stored at -80C until further usage. Mice were genotyped by PCR performed by Transnetyx Inc, Automated Genotyping Services. Experiments were all approved by the Danish National Ethics Committee and performed according to the European Convention (ETC 123 of 1986).

## 5xFAD mice

Transgenic heterozygote male 5xFAD mice (TG(APPSwFlLon,PSEN1*M146L*L286V)6799Vas, B6/SJLF1J, Jax strain: 006554) and wild-type females (B6/SJLF1J, Jax strain: 100012) were purchased from Jackson Laboratories (Bar Harbor, ME, USA). Breeding colonies were maintained at Department of Drug Design and Pharmacology, University of Copenhagen. Mice were housed together in individually ventilated cages in a specific pathogen-free, humidity and temperature-controlled facility (12h light/dark cycle) with free access to water and chow. Heterozygote 5xFAD mice and wild-type littermates (controls) were used at 6 months of age, corresponding to an intermediate disease stage.^3^ As 5xFAD mice display sex-specific variations in brain amyloid pathology^3^ only female mice were included in this study. Mice were genotyped by a PCR protocol as described in.^4^ Female heterozygote 5xFAD and wild-type littermates were euthanized one at a time by cervical dislocation. The brain was quickly excised from the cranial vault and snap-frozen by transfer to finely pulverized dry ice. Once frozen, the brain was stored at -80˚C, before further analysis. Experiments were all approved by the Danish National Ethics Committee and performed according to the European Convention (ETC 123 of 1986).

## ArcSwe mice

Male and female ArcSwe mice were obtained by in-house breeding on a C57bl/6 background.^5^ The mice were housed in groups at an animal facility at 20-22˚C at Uppsala University with access to food and water *ad libitum* on a 12-h light/dark cycle. ArcSwe mice and WT littermates of the same ages were euthanized at the age of 18 months. Mice were anesthetized with 2.7-3.2% isoflurane (Baxter Medical AB, Kista, Sweden) followed by intracardical perfusion for 2 min. After perfusion, the brain was removed from the cranium and snap frozen on dry ice. Mice were genotyped by PCR performed according to previously described procedure.^6^ All mice used in this study were maintained under protocols approved by the Uppsala County Animal Ethics board, and followed the rules and regulations of the Swedish Animal Welfare Agency (approval number C17/14).

## Pretargeted Autoradiography

Pretargeted autoradiography was performed according to previously published procedure.^2^

Frozen brains of previously described 5xFAD, APP/PS1 and Tg-ArcSwe mice were cut into halves along the sagittal symmetry plane. Each half was mounted, lateral side up, on the cryostat sample holder pretreated by Tissue-Tek OCT Compound, and fixed by freezing in the cryostat. Brains were cut at −22 °C into 20 μm thick sagittal slices using a Leica microtome, and the slices were thaw-mounted on Superfrost (70 × 22 mm, Fischer) adhesive slides. Only slices containing both cortex (specific binding) and cerebellar regions (representing nonspecific binding) were used. The slices were allowed to dry and were then put into storage boxes and kept at −80 °C until they were used.

On the day of the experiment, the frozen slides with mounted brain sections were allowed to come to room temperature for 30 min. Afterwards, a PAP pen was applied around the sections and 600 μL 1% BSA in PBS (pH 7.4) with 0.05% Tween 20 was added and incubated at room temperature for 30 minutes in incubation boxes. Afterwards, the BSA solution was removed and 600 μL of PBS (pH 7.4) with 0.05% Tween 20 was applied and incubated at room temperature for 5 minutes. The preincubation buffer was removed and 600 μL of TCO-3D6 (0.06 μL/mL and 0.006 μL/mL) in PBS (pH 7.4) with 0.05% Tween 20 was applied and incubated overnight at room temperature in a moist incubation box. On the second day, the TCO-mAb solution was removed and the slides were washed in cold PBS (pH 7.4) (3x15 min) and cold demineralized water (1x30 sec). Then, 600 μL [^111^In]**1** (5 nM and 20 nM) solution was applied to all sections and incubated for 60 minutes. Afterwards, the radioactive solution was removed and the slides were washed in cold PBS (pH 7.4) (3x5 min) and demineralized water (1x30 sec) and dried by a flow of compressed air. A calibration curve with dilutions of [^111^In]**2**  - 40nM, 20nM, 6.7nM, 2.2nM, 0.74nM, 0.25nM and 0.12nM – was prepared. After drying the plates, the slides were exposed on phosphor storage screens for 1-12 hours, depending on the amount of radioactivity on the slide. Afterwards, the phosphor storage screens were read by a Cyclone Storage Phosphor System (Packard Instruments Co). Quantification of plate readings was done with Optiquant software (version 3.00, Packard Instruments Co) and ImageJ by drawing regions of interest on the cortex and cerebellum manually according to previously described protocol.^2^ Data was processed in Excel and cortex/cerebellum imaging contrast ratios were calculated.^7,8^

# References

1. Lewis JS, Windhorst AD, Zeglis BM. *Radiopharmaceutical Chemistry*. *Neuromethods* (Springer, 2019). doi:10.1007/7657-2012-41

2. Shalgunov V, Lopes van den Broek S, Anderson IV, Vázquez RG, Raval N,Palner M, *et al.* Pretargeted Imaging Beyond the Blood-Brain Barrier. *ChemRxiv* **Cambridge**, (2022) doi: 10.26434/chemrxiv-2022-gj597-v2

3. Oakley H, Cole SL, Logan S, Maus E, Shao P, Craft J, *et al.* Intraneuronal beta-Amyloid Aggregates, Neurodegeneration, and Neuron Loss in Transgenic Mice with Five Familial Alzheimer’s Disease Mutations: Potential Factors in Amyloid Plaque Formation. *J. Neurosci.* **26**, 10129–10140 (2006).

4. Andersen JV, Skotte NH, Christensen SK, Polli FS, Shabani M, Markussen KH, *et al.* Hippocampal disruptions of synaptic and astrocyte metabolism are primary events of early amyloid pathology in the 5xFAD mouse model of Alzheimer’s disease. *Cell Death Dis.* **12**, 954 (2021).

5. Olsen M, Aguilar X, Sehlin D, Fang XT, Antoni G, Erlandsson A, *et al.* Astroglial Responses to Amyloid-Beta Progression in a Mouse Model of Alzheimer’s Disease. *Mol. Imaging Biol.* **20**, 605–614 (2018).

6. Pagnon de la Vega, M. Characterization of the novel “Uppsala mutation” causing a familial form of early onset Alzheimer’s disease. (Uppsala University, 2021). doi:http://uu.diva-portal.org/smash/record.jsf?aq2=%5B%5B%5D%5D&c=9&af=%5B%5D&searchType=LIST_COMING&sortOrder2=title_sort_asc&query=&language=en&pid=diva2%3A1571680&aq=%5B%5B%5D%5D&sf=all&aqe=%5B%5D&sortOrder=author_sort_asc&onlyFullText=false&noOfRows=50&dswid=3520

7. Raval RN, Johansen A, Donovan LL, Ros NF, Ozenne B, Hansen HD, *et al.* P.804A single dose of psilocybin increases synaptic density and decreases 5-HT2A receptor density in the pig brain. *Eur. Neuropsychopharmacol.* **40**, S453–S454 (2020).

8. Griem-Krey N, Klein AB, Herth M, Wellendorph P. Autoradiography as a Simple and Powerful Method for Visualization and Characterization of Pharmacological Targets. *J. Vis. Exp.* (2019). doi:10.3791/58879
